# Crystallographic Engineering of Spin Transport in Antiferromagnetic NiO Thin Films

**DOI:** 10.1021/acsnano.5c06120

**Published:** 2025-09-03

**Authors:** Shoulong Chen, Alberto Pomar, Lluis Balcells, Zorica Konstantinovic, Bernat Bozzo, Carlos Frontera, Cesar Magen, Narcis Mestres, Benjamin Martinez

**Affiliations:** † Instituto de Ciencia de Materiales de Barcelona. ICMAB-CSIC. Campus Universitario UAB, Bellaterra 08193, Spain; ‡ Center for Solid State Physics and New Materials, Institute of Physics Belgrade, University of Belgrade, Belgrade 11000, Serbia; § Instituto de Nanociencia y Materiales de Aragón (INMA), CSIC-Universidad de Zaragoza, Zaragoza 50009, Spain

**Keywords:** spin pumping, spin currents transmission, inverse spin Hall effect, complex oxides heterostructures, antiferromagnets

## Abstract

In this work, we investigate how the crystallographic growth direction influences spin current transmission in antiferromagnetic (AF) NiO thin films. By manipulating epitaxial growth, we explored the spin transport characteristics in La_2/3_Sr_1/3_MnO_3_/NiO/Pt heterostructures grown on top of (001)- and (111)-oriented SrTiO_3_ substrates, varying the NiO barrier thickness (t_NiO_). Spin currents were generated via spin pumping (SP), and detection was done by the inverse spin Hall effect (ISHE). X-ray diffraction and high-resolution electron microscopy techniques confirmed high-quality epitaxial films with nearly atomically sharp interfaces and similar dislocation distributions, irrespective of the growth direction. Nevertheless, epitaxially engineered (111) heterostructures exhibited superior spin transport properties, including lower magnetic damping (α), longer spin diffusion lengths (λ_Sd_), and higher spin mixing conductance (*g* ^↑↓^). The temperature dependence of the ISHE voltage signal (V_ISHE_) also showed orientation-dependent behavior: while (001)-oriented samples followed a monotonic trend, (111)-oriented samples displayed a peak that shifted to higher temperatures with increasing t_NiO_, associated with the emergence of AF ordering. Moreover, (111)-oriented samples demonstrated notable spin current amplification at room temperature, peaking at t_NiO_ ≈ 1 nm before decaying quasi-exponentially, indicative of spin diffusion-mediated conduction. Although the spin diffusion length in (111)-oriented samples was roughly double that of their (001)-oriented counterparts, it was still too short to be explained by angular momentum transport by mobile antiferromagnons through NiO. Instead, these findings point to a mechanism involving magnetic correlations and short-range thermal magnons. The superior spin transport properties and the enhanced spin conduction in (111)-oriented samples are primarily attributed to a synergistic combination of interfacial and dynamic effects, a more favorable Néel vector alignment and distinct interface symmetry, which can enhance spin-Hall effects or enable different spin textures. Overall, this study underscores the pivotal role of the Néel vector and crystallographic orientation in AF spin transport, providing valuable insights for the design and optimization of spintronic devices.

## Graphical abstract

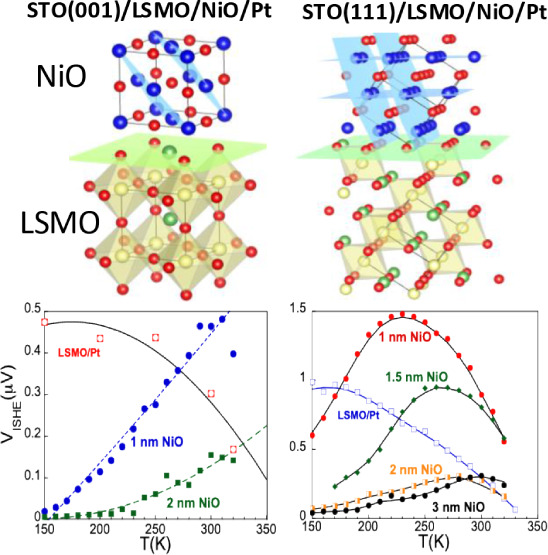

## 1 Introduction

Research in spintronics has traditionally focused on ferromagnetic (FM) materials due to their inherent properties, which make them highly suitable for technological applications. Among these properties, the existence of spontaneous magnetization is particularly significant, as it allows for direct manipulation of magnetic states through relatively weak external magnetic fields or electric currents. This has facilitated the development of advanced technologies, such as magnetoresistive random-access memories (MRAM) and high-performance magnetic sensors.^1,2^ In contrast, antiferromagnetic (AF) materials have historically been relegated to secondary, passive roles.^3^ However, recent breakthroughs have shown that AFs are far more versatile than previously thought. Recent experiments have shown that different AF insulator systems, including oxide materials^4–6^ and even 2D-semiconductor materials,^7,8^ allow very efficient spin current transmission while blocking charge conduction. Even more, spin current enhancement has also been observed in some cases.^9–11^ Furthermore, electrical switching, by using Néel spin–orbit torque, was demonstrated in metallic AFs,^12,13^ as well as in insulating AFs via spin torque.^10,14,15^ These findings, combined with the intrinsic properties of AFs, have promoted a strong interest in these materials as active components in spintronic devices.^16^

Although AFs lack spontaneous magnetization, they offer several unique advantages over their FM counterparts. They exhibit remarkable stability against external magnetic noise, generate no stray fields, and possess ultrafast dynamics in the terahertz range.^17,18^ These features make them ideal candidates for the next generation of spintronic devices, particularly in high-speed, low-power applications.

One of the most immediate and promising applications of AFs is their role as insertion barriers in FM/nonmagnetic (NM) bilayers. When used for such applications, AFs significantly affect interfacial spin transparency (T_int_), improving the efficiency of spin transfer between layers.^19–23^ Nonetheless, critical aspects related to the microstructure of AF layers, such as whether they are polycrystalline or epitaxial, as well as their crystallographic orientation, remain poorly understood. These factors are expected to play a significant role in facilitating long-range AF ordering and influencing interfacial interactions, as well as spin current amplification. However, their exact impact on spin transport and interfacial dynamics remains largely unexplored and requires further investigation to be fully understood.^24,25^

Among the insulating AF materials, NiO, with a bulk Néel temperature of T_N_ = 523 K, has been one of the most studied because of its simple crystallographic structure and its, a priori, relatively simple AF ordering.^26,27^ NiO crystallizes in a face-centered cubic (FCC) structure, where the Ni^2+^ cations occupy the octahedral sites and the O^2 –^ anions are positioned in the tetrahedral sites of the lattice. Below T_N_, Ni^2+^ magnetic moments order antiferromagnetically following an alternating up–down pattern along any ⟨111⟩ crystallographic direction. Consequently, the Ni^2+^ cations form two interpenetrating sublattices, with their spins oriented oppositely and lying perpendicular to the propagation vector of the AF ordering.^28^ However, things are not so simple and in bulk NiO crystals magnetostriction, due to FM ordering inside the planes, causes cell distortion below T_N_ (NiO undergoes a weak cubic-to-rhombohedral distortion (*R*3̅*m*)) creating a complex structure of the so-called T domains (regions with different orientations of the rhombohedral distortion axis) and S domains (areas within each T-domain where the antiferromagnetic spin axis adopts different orientations in the corresponding {111} plane) with 12 possible spin orientations.^26,27^

Although in thin films the number of possible domain orientations is significantly reduced,^29,30^ determining the precise orientation of the Néel vector (which describes the AF ordering) is very challenging. Understanding and controlling the Néel vector orientation is crucial for applications in AF spintronics, as it directly impacts the magnetic anisotropy, domain structure, and switching behavior of NiO-based devices.^31^ Previous studies on spin transport in NiO films with different orientations are limited.^23^ Notable differences in spin diffusion length between (001)- and (111)-oriented NiO films were reported. However, samples used in this study were grown on different substrates, resulting in notable differences in lattice mismatch and structural strain that can significantly alter the interfacial matching conditions and consequently influence spin current diffusion.

In this work, we investigate how an AF material, used as an insertion barrier, modifies interfacial properties in FM/NM bilayers and identify mechanisms to optimize pure spin current transmission for potential spintronic applications. To study the effect of microstructure and growth crystallographic direction on spin current transmission epitaxial La_2/3_Sr_1/3_MnO_3_ (LSMO)/NiO/Pt heterostructures with different NiO layers thickness, t_NiO_, were epitaxially grown on SrTiO_3_ (STO) (001) and (111) oriented substrates by RF sputtering. LSMO is the archetypical half-metallic FM manganese perovskite with a Curie temperature, T_C_, well above room temperature and strong magnetoresistance, whose effectiveness as a spin injector has been previously tested.^6,32,33^ Pure spin currents were generated in the LSMO layer by ferromagnetic resonance (FMR) excitation, enabling spin pumping across the LSMO/NiO interface. The spin angular momentum current propagated through the NiO layer and was detected in the Pt layer by measuring the transverse voltage signal (V_ISHE_) resulting from the inverse spin Hall effect (ISHE). Structural characterization using X-ray diffraction (XRD) and various scanning transmission electron microscopy (STEM) techniques evidenced that LSMO grew epitaxially, cube on cube, onto the STO substrates. Similarly, NiO and Pt layers also exhibited high-quality epitaxial growth with excellent crystallinity. Notably, NiO layers were homogeneous and continuous down to values of t_NiO_ of about 1 nm, a significant improvement over polycrystalline NiO samples.^33^

These films and heterostructures exhibit excellent crystallinity, no detectable impurity phases, and exceptionally flat surfaces, as confirmed by atomic force microscopy (AFM). Despite a 7% lattice mismatch between LSMO and NiO, interface dislocations accommodate the strain, allowing epitaxial growth of NiO.

Our study reveals that epitaxial engineered (111)-oriented samples exhibit optimized magnetic characteristics, including reduced Gilbert damping (α), narrower ferromagnetic resonance (FMR) line widths, larger effective spin-mixing conductance $\left(G_{eff}^{↓↑}\right)$ and enhanced spin current conduction, even at room temperature (RT), thus above the Néel temperature (T_N_). The temperature dependence of the transverse voltage (V_ISHE_), generated by inverse spin Hall effect (ISHE) shows clearly different temperature dependencies. To further explore spin conduction mechanisms, we examine the dependence of the V_ISHE_ signal on t_NiO_, finding a maximum at t_NiO_ ≈ 1 nm, followed by a quasi-exponential decay. This behavior, observed independently of crystal orientation, suggests a spin diffusion-mediated transport mechanism with spin diffusion length (λ_Sd_) values that depend on the crystal growth direction. The extracted values of λ_Sd_ suggest a conduction mechanism dominated by magnetic correlations and short-range thermal magnons. Our results also suggest that the observed ISHE signal amplification in the (111)-oriented may result from a synergistic combination of interfacial (enhanced spin transparency due to reduced spin backflow and spin memory loss) and dynamic effects (orientation-enhanced spin-wave transport efficiency due to a favorable orientation of the Néel vector and dynamic spin current enhancement mediated by AF fluctuations near the Néel temperature). On the other hand, our findings highlight the critical role of the Néel vector orientation and interface symmetry in enhancing spin transport efficiency. The superior spin conduction observed in (111)-oriented samples indicates that this crystallographic orientation is particularly favorable for optimizing spintronic devices based on AF materials.

## 2 Results and Discussion

XRD data reveal that the NiO layers are slightly strained in all cases, irrespective to the crystallographic growth direction (see Figure 1), with a larger unit cell volume compared to the bulk material (a = 4.18 Å). For the (001) oriented films, the pseudocubic lattice parameters are a = 4.182 Å and c = 4.253 Å, indicating a slight out-of-plane elongation of the NiO unit cell. In the case of the films grown along the (111) direction, analysis of the {200} peak positions suggest the formation of a hexagonal unit cell with parameters a_h_ = 5.897 Å and c_h_ = 7.400 Å, which would correspond to pseudocubic cell parameters of a_h_/√2 = 4.170 Å and c_h_/√3 = 4.273 Å. This would imply a slightly higher in-plane compression and a slightly higher out-of-plane elongation compared to (001)-oriented films. The observed lattice mismatch between the LSMO and NiO in-plane parameters is huge (7%), leading to the formation of a large number of interface dislocations (see Figure S4). These dislocations help the accommodation of NiO crystal structure on top of LSMO films, allowing the epitaxial growth between them.

**Figure 1:**
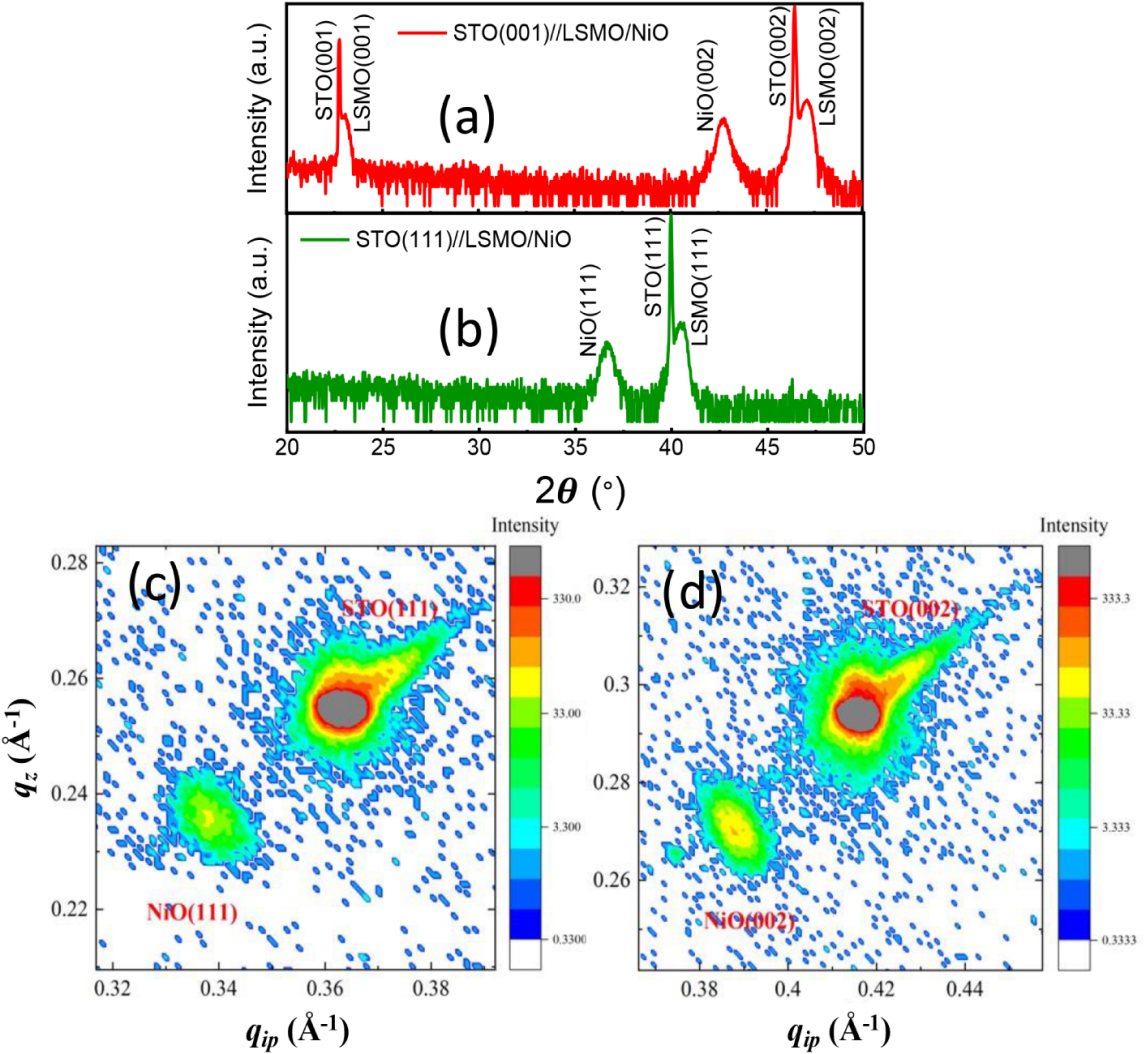
θ-2θ X-ray diffraction (XRD) spectra of STO(001)//LSMO/NiO­(25 nm) (a) and STO(111)//LSMO/NiO­(25 nm) (b) heterostructures. Reciprocal space maps (RSM) around the (111) peak of the STO(001)//LSMO/NiO­(25 nm) (c) and (002) peak of the STO(111)//LSMO/NiO­(25 nm) sample (d) are shown.

Aberration-corrected STEM was employed to analyze the structural properties of the heterostructures, with a particular focus on the NiO layers. Figure 2a–d presents high-angle annular dark-field (HAADF) images of samples featuring two nominal t_NiO_ of 1 and 2 nm. Due to the significant difference in the average atomic numbers of the three layers, the contrast has been artificially adjusted to allow simultaneous visualization of the NiO, Pt, and LSMO layers. The images clearly demonstrate the single-crystalline quality of the LSMO underlayer, as well as that of the NiO and Pt layers. Notably, the crystalline and interfacial quality of the LSMO/NiO/Pt heterostructures seems to be very much alike for both crystallographic orientations. Additionally, the measured NiO thicknesses align well with values estimated based on the evaporation rate, confirming the precision of the deposition process. The images also clearly show that the NiO layers are continuous, forming a conformal coating over the LSMO layer despite some surface roughness. Additionally, they provide evidence of largely epitaxial growth, in spite of a nominal lattice mismatch of about 7% for both substrate orientations, albeit with some mosaicity but without significant strain. This can be attributed to the formation of misfit dislocations at the LSMO/NiO interface, which help relieve the epitaxial strain. This is supported by high-resolution TEM images shown in the Supporting Information (See Figure S4). These images reveal a coherent cationic interface between NiO and LSMO along both the (001) and (111) crystallographic planes (Figure S4 a,b, respectively). Importantly, no transition layers or amorphous regions are observed at the interface.

**Figure 2:**
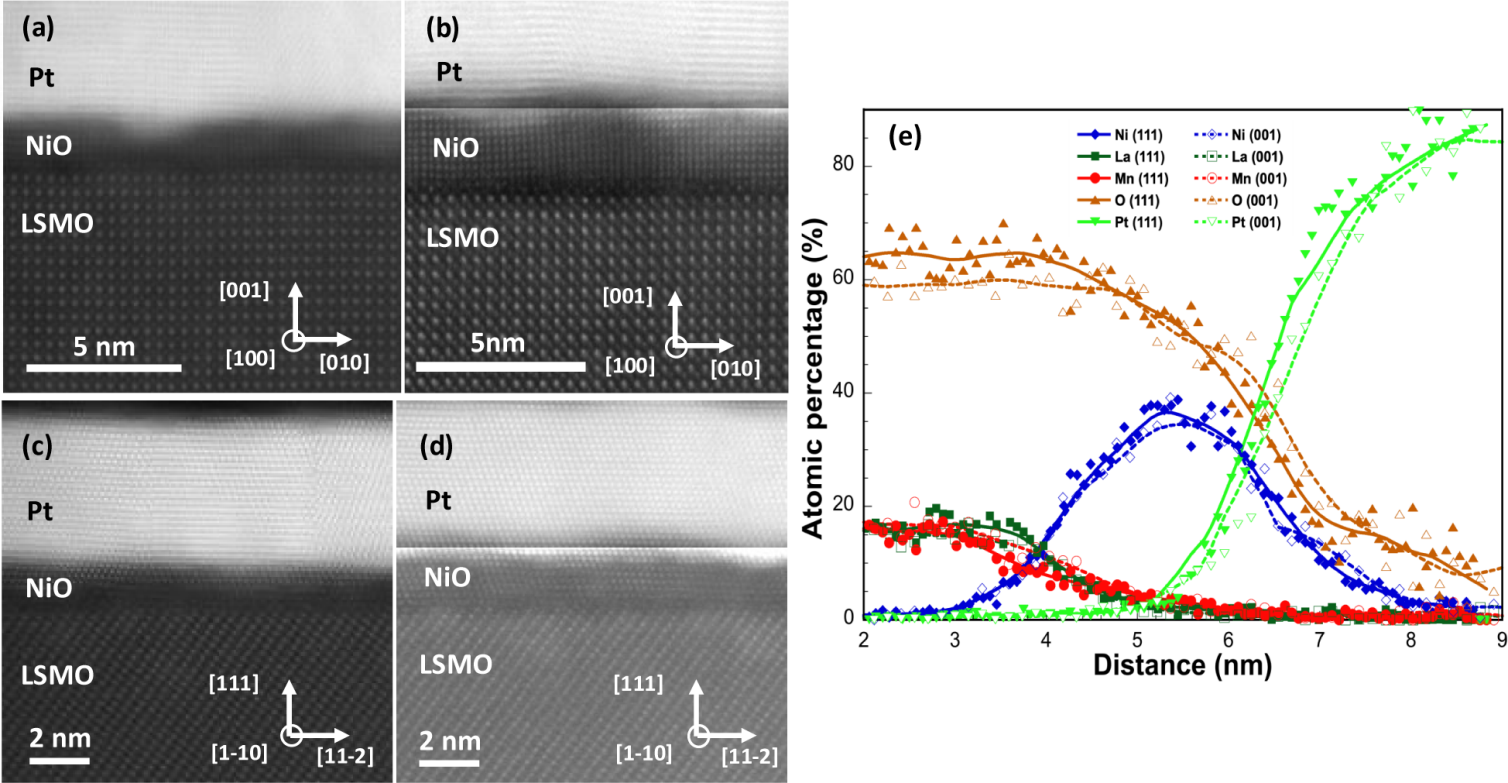
Atomic-resolution cross-sectional HAADF-STEM images of the different STO//LSMO/NiO/Pt heterostructures. The upper part of the figure corresponds to STO(001)//LSMO/NiO/Pt with nominal NiO layer thickness of: (a) 1 nm and (b) 2 nm. The lower part of the figure corresponds to STO(111)//LSMO/NiO/Pt with nominal NiO layer thickness of: (c) 1 nm and (d) 2 nm. The high quality of the LSMO films and abruptness of interfaces are evident, as well as the epitaxial character of both NiO and Pt layers. The contrast in the pictures has been artificially modified to visualize simultaneously the NiO, the Pt and the LSMO layers. (e) STEM-EDS chemical analysis of the LSMO/NiO/Pt trilayer for two samples with different crystallographic orientation: (001)-oriented (open symbols) and (111)-oriented (full symbols) with the same nominal NiO layer thickness (2 nm). Chemical line profiles, obtained by horizontal integration of ∼10 nm of the EDS maps across the interface, are shown for La, Mn, Ni, O and Pt atomic species (chemical line profiles for all the atomic species are shown in Figure S7). Continuous and dashed lines are guide to the eye.

On the other hand, Geometric Phase Analysis (GPA) of HAADF images confirmed that NiO layers on top of LSMO were slightly stressed showing a unit cell expansion respect to that of bulk, irrespective to the crystallographic growth direction (see Figures S5 and S6) consistent with the unit cell expansion observed in XRD measurements for a thicker NiO layer (25 nm).

Further insight into the quality of LSMO/NiO/Pt heterostructures was obtained through STEM-EDS chemical mapping (see Figure S7). Chemical line profiles, generated by horizontally integrating approximately 10 nm of the EDS maps across the interface, clearly demonstrate that the LSMO/NiO/Pt heterostructures exhibit high quality with sharp interfaces and relatively low atomic interdiffusion. Additionally, as shown in Figure 2e, the LSMO/NiO/Pt interfaces appear highly similar regardless of the crystallographic direction of growth. The NiO layer thickness extends to about 2.5 nm, while the Pt signal penetrates less than 1 nm into the NiO layer. A similar interdiffusion depth is observed at the LSMO/NiO interface. This can be attributed to minor interface roughness combined with the delocalization of the inelastic electron-specimen interaction responsible for X-ray generation. However, in strong contrast with NiO polycrystalline samples,^33^ no direct contact regions between LSMO and Pt are detected, even in samples with NiO layers as thin as 1 nm. This observation is also confirmed by HAADF images (see Figure 2a–d).

The DC magnetic properties of the heterostructures were studied by using SQUID magnetometry. In agreement with the very high crystalline quality of LSMO films a saturation magnetization of M_S_ ≈ 3.5 μ_B_/Mn, close to the theoretical value, and T_C_ slightly above 350 K were observed in all the samples irrespective of the growth crystallographic direction and the NiO layer thickness (see Figures 3a and S8). The AF ordering of NiO was examined through exchange bias field (H_EB_) measurements. However, it is important to note that since the T_C_ of the LSMO layer is lower than the T_N_ of the NiO layer, achieving a precise characterization remains challenging (see Figure S8).^34^ However, due to finite size effects, T_N_ decreases as t_NiO_ is reduced, providing an opportunity for a more accurate exchange bias measurements.^35,36^ For these measurements, samples were cooled from *T* = 400 K to the desired temperature under a 70 kOe field, after which the hysteresis loop was recorded. Using this protocol, the presence of exchange bias—indicating AF ordering in the NiO layer—was confirmed in STO//LSMO/NiO/Pt heterostructures with a 5 nm thick NiO layer irrespective to the crystallographic growth direction (see Figure 3b).

**Figure 3:**
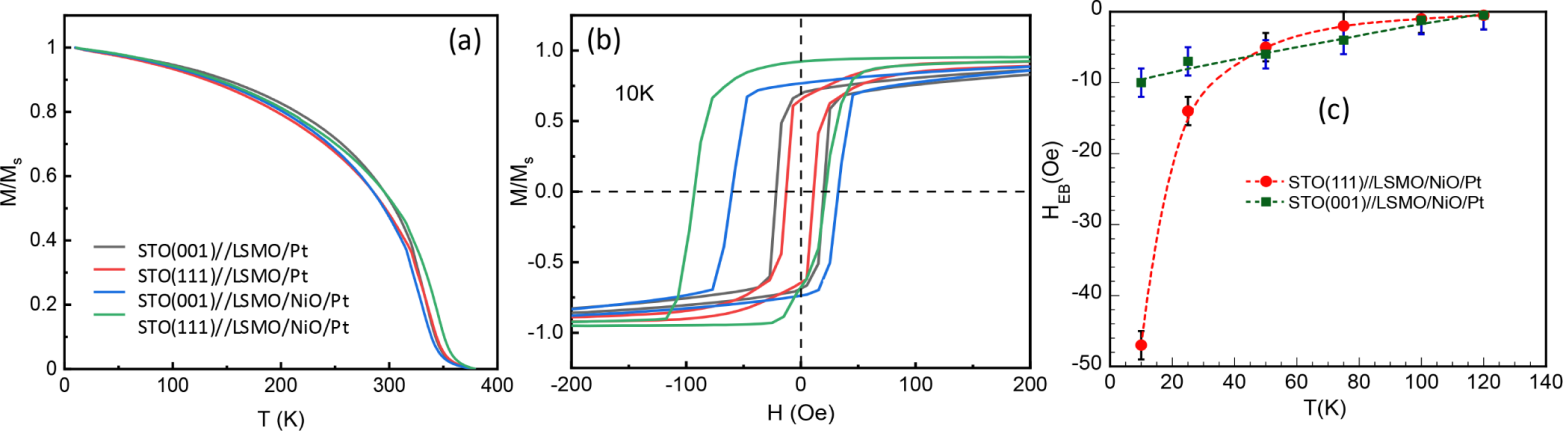
(a) Temperature-dependence of magnetization of LSMO films, with a nominal thickness of 15 nm, in STO//LSMO/Pt bilayers and in STO//LSMO/NiO/Pt trilayers featuring a 5 nm NiO layer, grown along the (001) and (111) crystallographic directions. The magnetization values have been normalized to their low-temperature values to make evident the identical behavior in all cases, regardless of the presence of the NiO layer. (b) Hysteresis loops at *T* = 10 K for STO//LSMO/Pt bilayers grown along the (001) and (111) crystallographic directions, compared to the corresponding loops for STO//LSMO/NiO/Pt trilayers with a 5 nm NiO layer. The emergence of the exchange bias field is clearly observed. (c) Temperature dependence of the exchange bias field in LSMO/NiO/Pt heterostructures for (001) and (111) crystallographic growth directions corresponding to a 5 nm thick NiO layer.

At low temperature, magnetic hysteresis loops, M­(H), are clearly shifted along the magnetic field axis for both crystallographic directions, thus demonstrating the presence of exchange bias and, consequently, the AF ordering of the NiO layer. Additionally, the temperature dependence of H_EB_, shown in Figure 3c, reveals that the blocking temperature, T_B_, characteristic of the FM/AF exchange bias interaction, is about 120 K for the 5 nm NiO samples regardless of the crystallographic growth orientation. Since T_B_ is a lower bound of T_N_, it is to be expected that T_N_ is above this value. The value obtained here for T_B_ is low compared to previous results^11^ and, as we will see later on, to our results obtained from V_ISHE_ measurements. This discrepancy could be related to the fact that the actual T_N_ of a 5 nm NiO layer may be well above room temperature and therefore, the measurement procedure would not be strictly correct since T_N_ would be clearly above T_C_.^37^ However, H_EB_ is larger in (111)-oriented samples, suggesting a stronger interfacial interaction in this case.

The dynamic magnetic properties of LSMO thin films and LSMO/NiO/Pt trilayers were investigated using FMR. To ensure that the quality of all LSMO samples was comparable (similar line width, ΔH), ΔH was measured by fitting the corresponding spectrum to a Lorentzian derivative for each sample prior to NiO and Pt deposition. To examine spin current transmission through the NiO layer a detailed analysis of spin injection and spin-charge conversion processes was performed as a function of t_NiO_ and temperature. For this purpose, FMR and ISHE measurements were conducted over a broad temperature range by sweeping the externally applied magnetic field, *H*, in the microwave frequency range of 2 to 16 GHz. The static external magnetic field was applied in-plane, aligned parallel either to the [100] or [11–2] substrate directions for samples grown along the (001) and (111) directions, respectively. Experimental differential absorption spectra at resonance for samples grown along (001) and (111) crystallographic directions, recorded at various temperatures, frequencies, and t_NiO_ values, were fitted to a Lorentzian derivative with both symmetric and antisymmetric contributions.^38^ From these fittings, ΔH and the resonance field (H_res_) were determined. These parameters are directly related to the magnetic properties of the samples through the Kittel equations.^39^

1 $$\begin{array}{l} f_{res}=\frac{\gamma }{2\pi }{\left(H_{res}+\frac{2K_{4}}{M_{s}}\right)}^{1/2}\cdot {\left(H_{res}+4\pi M_{eff}+\frac{2K_{4}}{M_{s}}\right)}^{1/2}\\ \Delta H=\Delta H\left(0\right)+\frac{2\pi }{\gamma }\alpha f_{res} \end{array}$$

Being *f*_*res*_ the resonance frequency, *K*_4_ the fourth-order magnetic anisotropy term and *M*_*eff*_ the effective magnetization (*4πM*_*eff*_ *= 4πM*_*S*_ – *2K*_2_ */M*_*S*_, where *K*_2_ is the second-order anisotropy term and *M*_*S*_ is the saturation magnetization of the LSMO film). *ΔH­(0)* stands for the inhomogeneous line width broadening. By applying the Kittel equations, the gyromagnetic ratio (γ), M_eff_, and Gilbert damping parameter (α) were estimated. The obtained values for M_eff_ and γ are consistent with those previously reported for LSMO films.^6,33^ At this stage, it is important to emphasize that α and *ΔH­(0)* in LSMO layers are notably lower when they are grown in the (111) orientation. In both (001) and (111)-oriented samples, the inhomogeneous line width broadening increases for thicker samples. This increase may be related to strain relaxation in thicker films leading to the formation of local defects. In both directions, an increase of α is observed after depositing NiO and Pt layers, as anticipated. Moreover, α clearly rises up with t_NiO_ and shows a clear trend toward saturation for thicknesses beyond approximately 4–5 nm (see Figure 4a). If we take the increase of damping relative to a 15 nm reference LSMO layer, Δα = α_Pt/NiO/LSMO_ – α_LSMO/Pt_, as an indicator of spin angular momentum flowing through the NiO layer into the Pt layer via spin pumping, then the real part of the spin mixing conductance (g^↑↓^) can be estimated using the following expression:^40,41^

2 $$\Delta \alpha =\frac{g\mu_{B}g^{↑↓}}{4\pi M_{s}t_{LSMO}}\left[1-\exp\left(-2t_{NiO}/\lambda_{Sd}\right)\right]$$

**Figure 4:**
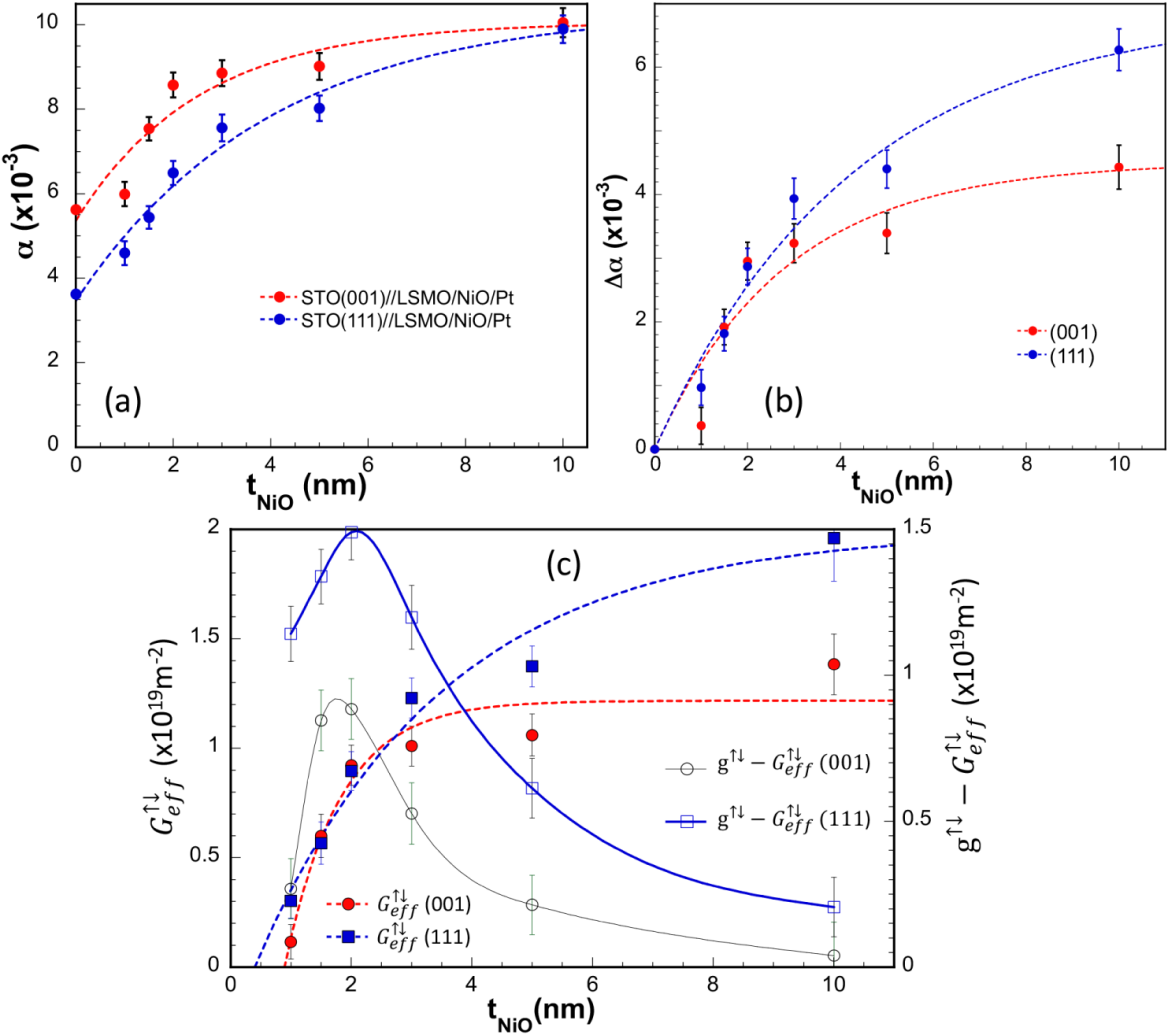
(a) Dependence of the Gilbert damping constant, α, on the NiO layer thickness at RT in STO//LSMO/NiO/Pt heterostructures grown along the (001) and (111) crystallographic directions. Dashed lines are guides to the eye. (b) Variation of α as a function t_NiO_ at RT relative to a LSMO­(15 nm)/Pt­(5 nm) reference layer. Lines correspond to the fitting according to eq 2 in the text. (c) Effective spin mixing conductance at RT as a function of the NiO layer thickness for samples grown along the two different crystallographic directions. The dashed lines serve as guides for the eye. The difference between the real part of the spin mixing conductance, $g^{↑↓}$ , and the effective spin-mixing conductance, $G_{eff}^{↓↑}$ , plotted on the right-hand axis, illustrates the significance of SBF effects.

Being λ_Sd_ the spin diffusion length and *t*_*LSMO*_ the thickness of the FM LSMO layer. Here it is assumed that the observed variation in α is mainly driven by changes in the NiO layer thickness. Strictly speaking, the exponential term should include the thickness of the spin sink, but in this case the Pt thickness is fixed. Additionally, spin accumulation within the NiO/Pt bilayer may lead to some spin backflow (SBF) into the LSMO layer, effectively reducing the spin pumping current.

The fitting of the experimental data to eq 2 (see Figure 4b) gives values of *g* ^↑↓^ at RT of 1.3 × 10^19^ m^–2^ and 2 × 10^19^ m^–2^ for the (001) and (111)-oriented samples, respectively. On the other hand, values of λ_Sd_ of 5.6 and 8.5 nm for the (001) and (111)-oriented samples respectively are estimated. The SBF can be accounted for by replacing *g* ^↑↓^ by an effective spin-mixing conductance, $G_{eff}^{↓↑}$ , that can be estimated as^4,6^

3 $$G_{eff}^{↑↓}=\Delta \alpha 4\pi M_{s}t_{LSMO}/\hbar \gamma$$

As shown in Figure 4c, the effective spin mixing conductance increases with NiO layer thickness and tends toward saturation for thicker NiO layers. While the overall values are comparable in both crystallographic orientations, the (001)-oriented samples reach saturation more quickly and at smaller thickness values suggesting a more pronounced interfacial spin transport efficiency in the (111) direction. On the other hand, the values obtained for $G_{eff}^{↓↑}$ are similar to those reported for other interfaces such as Pt/Py ( $G_{eff}^{↑↓}$ ≈ 2.1 × 10^19^ m^–2^)^42^ and Pt/NiO­(1 nm)/YIG ( $G_{eff}^{↑↓}$ ≈ 0.18 × 10^19^ m^–2^).^43^

By combining eqs 2 and 3, the variation of *g* ^*↑↓*^ at room temperature as a function of t_NiO_ can be determined, allowing for an estimation of the relevance of SBF effects. As illustrated in Figure 4c, interfacial spin losses, such as SBF, reach their maximum impact for t_NiO_ ≈ 2 nm. The observed behavior indicates that above a certain thickness, the additional NiO no longer affects the interfacial spin dissipation and a balance between spin current transmission, spin memory loss (SML) and SBF is reached (see Figure S10).

To determine how much of the theoretically available spin current is actually transmitted to the Pt layer the transverse voltage signal generated by ISHE in the Pt layer has been measured. Representative data of the measured transverse ISHE voltage signals are shown in Figure 5. As expected, according to the experimental setup (see Figure S3), the V_ISHE_ voltage signals are positive. Since these signals originate from the same magnetization dynamics that govern FMR, their line shape can also be accurately described by a Lorentzian function.

4 $$V_{ISHE}=\frac{V_{s}\Delta H^{2}}{\Delta H^{2}+4{\left(H-H_{\mathrm{res}}\right)}^{2}}+\frac{V_{\mathrm{as}}\Delta H\left(H-H_{\mathrm{res}}\right)}{\Delta H^{2}+4{\left(H-H_{\mathrm{res}}\right)}^{2}}$$

**Figure 5:**
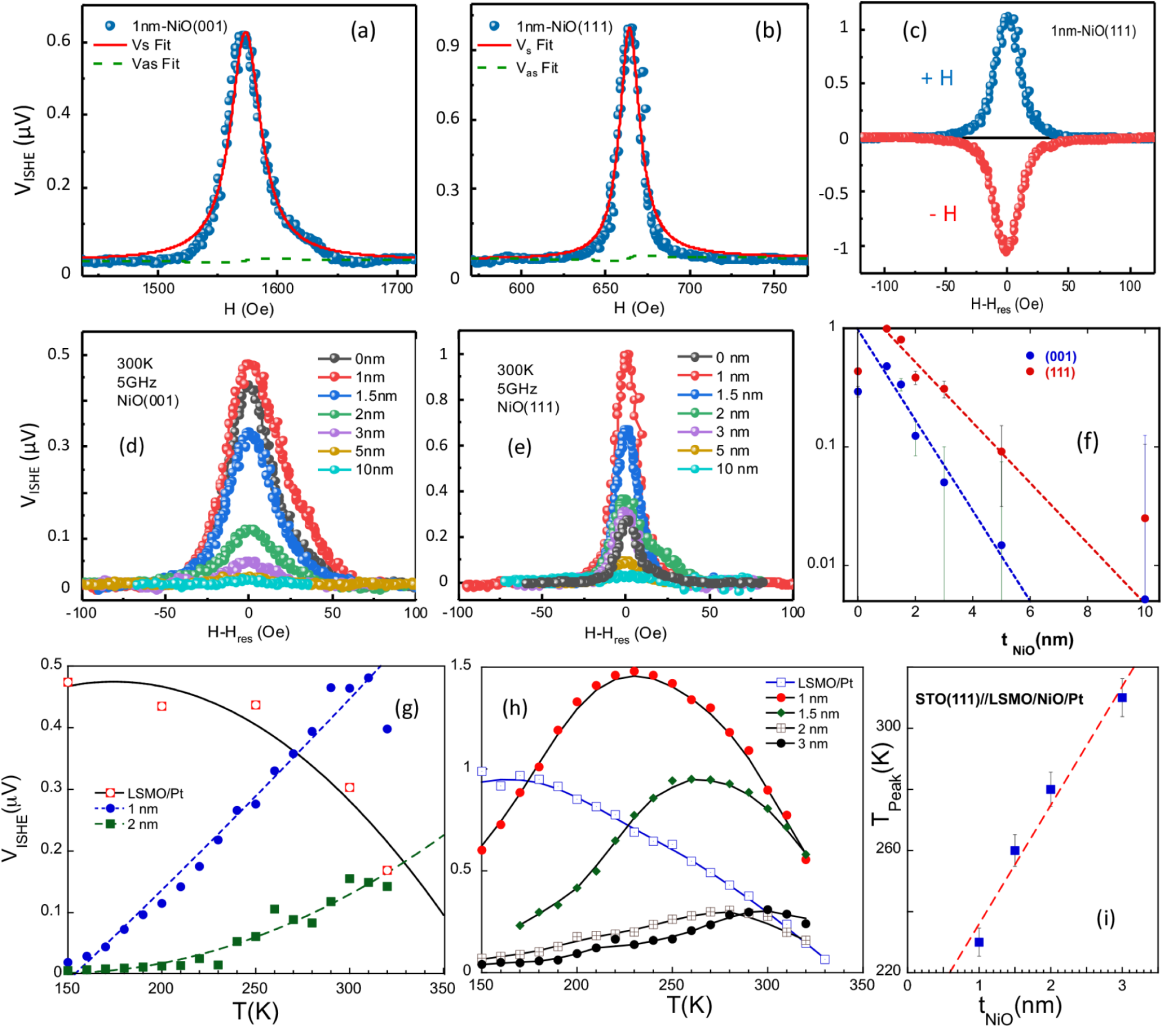
Transverse voltage signals (V_ISHE_) as a function of the magnetic field at room temperature (RT) corresponding to LSMO/NiO/Pt samples with t_NiO_ = 1 nm grown along the (001) (a) and (111) (b) crystallographic directions. The solid red line and dashed green line represent the symmetric and antisymmetric voltage components, respectively. (c) Transverse voltage signal measurements at RT at positive (blue) and negative (red) magnetic fields for an LSMO/NiO/Pt sample grown along the (111)-direction and t_NiO_ = 1 nm. Transverse voltage signal, V_ISHE_, as a function of NiO layer thickness, t_NiO_, measured at room temperature (RT) for samples grown along the (001) (d) and (111) (e) crystallographic directions. (f) V_ISHE_ signal variation with NiO layer thickness for both crystallographic orientations. Dashed lines represent exponential decay fits to the experimental data. Temperature dependence of the V_ISHE_ voltage signal as a function of the NiO layer thickness for (001) (g) and (111) (h) oriented samples. The V_ISHE_ voltage signal corresponding to a LSMO­(15 nm)/Pt­(5 nm) reference sample is also included for comparison. (i) Dependence of the peak temperature (T_peak_) on t_NiO_ for (111)-oriented samples for the low t_NiO_ range.

Where *V*_*s*_ and *V*_*as*_ stand for the symmetric and antisymmetric voltage amplitudes respectively, in analogy with the FMR signal. Additionally, both *ΔH* and *H*_*res*_ are the same as in FMR absorption. However, the relative magnitude of the symmetric and antisymmetric contributions often differs from that in FMR absorption. The ISHE transverse voltage signal is expected to exhibit a fully symmetric Lorentzian line shape.^44^ However, additional parasitic voltage contributions, such as spin rectification effects (SRE) or thermoelectric effects, may also be present.^45^ As shown in Figure 5a,b, V_ISHE_ is positive and almost fully symmetric, as expected, since SRE effects are likely negligible due to the high resistivity of the LSMO films and the insulating character of the NiO layers. Furthermore, Figure 5c presents V_ISHE_ measurements at a fixed frequency with the applied magnetic field direction reversed. It shows that the V_ISHE_ signals are inverted while maintaining the same magnitude. This, along with the negligible contribution from the antisymmetric component, indicates that thermal effects are insignificant and rules out any substantial thermoelectric contribution. As shown in Figure 4, increasing t_NiO_ promotes an increase of Δα and $G_{eff}^{↓↑}$ , which is, in turn, clearly reflected in the dependence of V_ISHE_ on t_NiO_, as depicted in Figure 5d,e. These figures present the dependence of the amplitude of the symmetric component of the V_ISHE_ signal, measured at RT, as a function of t_NiO_.

Several key observations can be drawn from Figure 5d,e. First, the voltage values in samples grown along the (111) direction are nearly twice as large as those in samples grown along the (001) direction. In contrast, the line width is significantly broader in the (001)-oriented samples, consistent with the previously observed behavior of α. Additionally, a clear signal amplification effect is observed, extending up to t_NiO_ ∼ 3 nm for (111)-oriented samples. For (001)-oriented samples, this amplification is considerably weaker and is only noticeable at t_NiO_ = 1 nm close to RT. This is in agreement with other previously published results,^9–11^ but in contrast with our previous results in LSMO/NiO/Pt systems with polycrystalline NiO layers of similar thickness, where no amplification was observed.^33^ The V_ISHE_ signal amplitude has the maximum for t_NiO_ = 1 nm and then decreases progressively as t_NiO_ increases up to the detection levels of the experimental setup (≈20 nV) for t_NiO_ ≥ 10 nm due to spin depolarization. The dependence of the V_ISHE_ signal amplitude on t_NiO_ is better illustrated in Figure 5f. A quasi-exponential decay with increasing t_NiO_, indicative of diffusive spin transport through the NiO layer, is observed.

By fitting the V_ISHE_ vs t_NiO_ experimental data with an exponential decay model values of λ_Sd_, of 2.3 and 3.4 nm are obtained for the (001)- and (111)-oriented samples, respectively (see Figure 5f). The discrepancy between these λ_Sd_ values and those derived from the increase in α using eq 2 may be attributed to the fact that in this latter approach, the values are determined from the direct reduction of the spin current, thus including contribution from spin current dissipation into the Pt layer. As far as this point is concerned, there is an extensive collection of λ_Sd_ values in the literature ranging from about 1.5 nm to several tens of nm,^9^,^21–23^ and although no clear trend has been identified in polycrystalline or epitaxial samples, some notable differences have been reported in single-crystalline NiO thin films with different spin transmission lengths along the NiO [001] and NiO [111] crystallographic directions.^23^ In our case, slightly different values are found for (001) and (111)-oriented samples but very similar to those reported in our previous work for polycrystalline NiO films.^33^ Hence, the significant variation in λ_Sd_ values indicates that, despite extensive research, it remains unclear which mechanisms govern spin current conduction through the NiO layer.

Moreover, the role of interface microstructure in modulating factors such as SBF and SML, key contributors to the interfacial spin transparency (T_int_), is still not fully understood. However, clearly different behavior between (001) and (111)-oriented samples is found when the temperature dependence of the V_ISHE_ is analyzed. The temperature dependence of the V_ISHE_ voltage signal is shown in Figure 5g,h. V_ISHE_ signal in (001)-oriented samples decreases monotonically with decreasing the temperature. This monotonic decrease is attributed to the decrease in the resistivity of Pt (see Figure S9c), the increase in the magnetization damping in LSMO and the increase of the AF interaction hardness, as was already observed in NiO polycrystalline samples.^33^ However, unlike the behavior observed in the polycrystalline samples, a small amplification of the V_ISHE_ signal is detected at temperatures close to RT for the t_NiO_ = 1 nm sample as can be appreciated in Figure 5g. For thicker NiO layers no signal enhancement is found when compared with the V_ISHE_ signal of the STO (001)//LSMO/Pt reference sample. Worth to mention here that, according to the temperature dependence of the magnetization of the LSMO (see Figure 3a), a progressive reduction of the V_ISHE_ voltage signal should be expected as shown for the LSMO/Pt bilayer in Figure 5g,h.

In contrast, (111)-oriented samples exhibit a pronounced temperature dependence and sensitivity to the NiO layer thickness, characterized by a well-defined broad peak (see Figure 5h). As t_NiO_ increases from 1 to 3 nm, the peak temperature, T_Peak_, gradually rises, while the peak height undergoes a sharp and nonmonotonic change from about 1.5 μV to 0.29 μV. Furthermore, as illustrated in Figure 5i, T_Peak_ increases linearly with t_NiO_ (obviously, this linear dependence is only valid in the range of a few nm (t_NiO_ ≤ 6–8 nm) since T_N_ will quickly tend to the bulk value for large NiO thicknesses). Below T_Peak_, V_ISHE_ progressively decreases since long-range AF order suppresses incoherent magnons, reducing spin transport efficiency. A similar trend has recently been observed in other oxide systems (YIG/NiO/Pt and YIG/CoO/Pt) using spin Seebeck effect,^11^ or spin pumping^46^ as the spin current injection method, and even in metallic systems (IrMn/Cu/NiFe) also via spin pumping.^47^ The peak in V_ISHE_ has been identified as the onset temperature of the AF ordering of the NiO layer that, due to finite size effects,^11,29^ is substantially less than the T_N_ of bulk material. These findings differ from our previous estimation of T_B_ (∼120 K for a 5 nm NiO layer, regardless of crystallographic orientation) based on exchange bias measurements. V_ISHE_ measurements on (111)-oriented samples suggest considerably higher T_N_ temperatures, even for much thinner NiO layers. Based on the results shown in Figure 5i, a 5 nm NiO layer would be expected to show a T_N_ close to 400 K. Meanwhile, the temperature dependence of V_ISHE_ signals in the (001)-oriented samples do not show any indication of the appearance of the AF ordering for any value of t_NiO_. This is an intriguing result since the detection of the appearance of AF order in ultrathin NiO layers by V_ISHE_ measurements has been reported previously in both polycrystalline and epitaxial samples.^11,46,47^ Nonetheless, literature reports are contradictory, with some studies reporting no observable peak in the V_ISHE_ amplitude as temperature varies for NiO samples of similar thickness, regardless of whether they are polycrystalline or epitaxial.^33,48^ Discrepancies between studies in the literature can often be attributed to variations in sample preparation techniques, equipment, and growth conditions, leading to differences in microstructure. However, in the present study, all samples were fabricated using the same equipment and identical growth conditions, with the only variable being the crystallographic orientation in which they were grown. Furthermore, high-resolution electron microscopy techniques reveal that the samples exhibit a highly similar, high-quality microstructure with identical interfacial characteristics (see Figure 2). Therefore, to explain the observed differences in magnetic and spin transport properties between samples grown in different crystallographic orientations, it is essential to consider the specific microstructure, atomic stacking sequence and electronic structure.

At any oxide interface, charge transfer between the materials leads to a significant redistribution of electronic states. In the LSMO/NiO system, the difference in electronic configuration and work function between doped FM LSMO and AF insulating NiO should drive notable charge transfer, triggering orbital reconstruction that in turn alters the local magnetic and transport properties and this might be uniquely influenced by the crystallographic orientation (see Figure 6).^49^ Epitaxial strain is another key factor, however, according to XRD and GPA analysis, in the present case there are no so much differences between (001) and (111)-oriented samples in this regard. Related to this, as mentioned in the introduction, due to magnetostriction inside the FM {111} planes of NiO, below T_N_ a complex structure of T and S domains appears (see Figure S11).^27,28^ The combination of T and S domains results in 12 possible spin orientations, which strongly affect magnetic, in particular the Néel vector orientation, and electronic properties.

**Figure 6:**
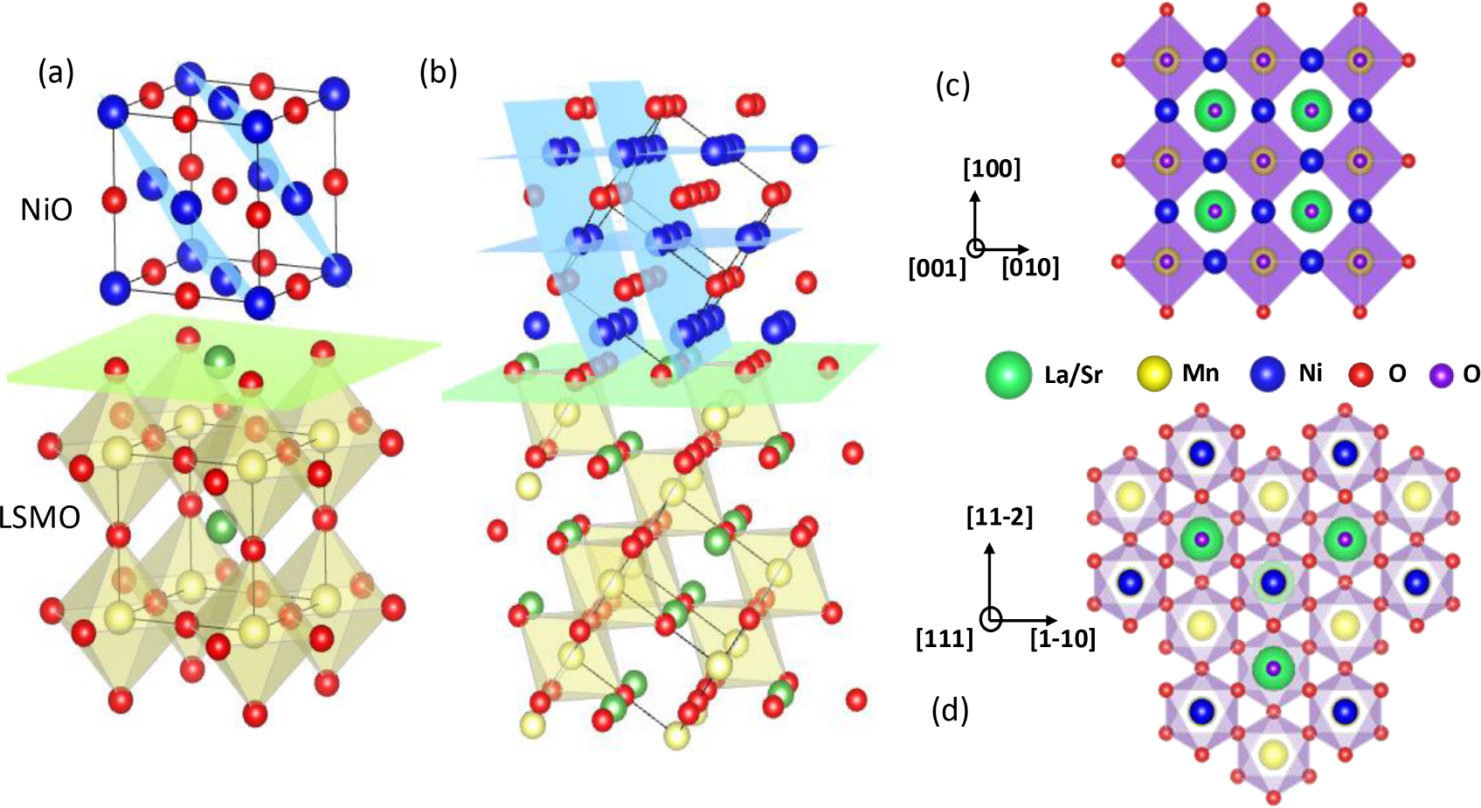
Sketch illustrating the NiO/LSMO bilayers grown along the (001) (a) and (111) (b) crystallographic directions. The epitaxial growth is assumed to be dictated by the anionic stacking of the face-centered cubic (fcc) NiO oxygen lattice atop the close-packed (La,Sr)O planes of LSMO. For simplicity, pseudocubic structures are depicted, disregarding lattice distortions and octahedral tilting. In both cases, a (La,Sr)­O-terminated interface layer (green plane) is imposed. The antiferromagnetic ordering easy planes, {111}, of NiO are represented in blue. Notably, for (111)-oriented films, one T-domain lies parallel to the substrate. In contrast, for (001)-oriented films, all magnetic domains are oriented out-of-plane. The atomic species are color-coded as follows: Ni (blue), O (red), Mn (yellow), and La/Sr (green). On the right side, the top-view schematics of NiO/LSMO(001) (c) and NiO/LSMO(111) (d) heterostructures are displayed, highlighting the respective Ni–O–Mn bonds. For clarity, oxygen atoms in LSMO and NiO are represented as red and purple spheres, respectively.

The relative orientation of the Néel vector with respect to the injected spin polarization is critical since only spin parallel or antiparallel to the Néel vector can be carried by magnons in antiferromagnetic insulators.^4,50^ When the Néel vector is favorably aligned, the coupling between the spin current and the AF ordering is enhanced, leading to more efficient magnon generation, and this might result in an amplification of the spin current signal. In bulk NiO, the spins are aligned along the ⟨111⟩ crystallographic directions. According to this, even structural strain might have some influence, in (001)-oriented NiO films, the ⟨111⟩ easy planes are oblique to the film plane and to the spin current injection direction. This orientation is canted by approximately 36° toward the out-of-plane direction compared to the bulk ⟨11–2⟩ directions.^31^ However, in (111)-oriented NiO films, one of the ⟨111⟩ easy planes is parallel to the film plane. This might lead to a simpler domain structure where a significant portion of the film has the Néel vector aligned along this in-plane axis and perpendicular to the spin current injection direction, i.e., parallel to the injected spins, thus favoring spin current transmission and a more efficient magnon generation which can be of great help in the spin current amplification process. This also promotes a longer spin diffusion length, allowing longer propagation distances. In contrast, the weaker coupling between the spin current and the AF ordering in (001)-oriented samples, stemming from the misalignment of the Néel vector and the spin current direction, likely accounts for the monotonic temperature dependence of the V_ISHE_ voltage, showing no clear indication of AF onset.

Additionally, the proximity of LSMO and NiO at the interface might give rise to magnetic proximity effects. Exchange bias, is a manifestation of this and, since it implies the interaction between FM ordered spins in LSMO layer and AF spins in NiO layer, it should be sensitive to the magnetic domain configuration of the NiO. In fact, as shown in Figure 3c at 10 K, the (111)-oriented interface exhibits a larger H_EB_ than the (001)-oriented one, indicating stronger interfacial exchange coupling. In an exchange-biased system, it is well-known that the H_EB_ depends on the thickness of the FM layer, t_FM_, according to H_EB_ = *J*_*K*_/M_S_t_FM_,^51^ being *J*_*K*_ the unidirectional anisotropy. In this context, *J*_*K*_ represents the effective interfacial exchange energy density, i.e., it quantifies the strength of the exchange coupling between the FM and AF layers and therefore, a larger *J*_*K*_ implies a stronger exchange interaction at the FM/AF interface. In particular, the calculated *J*_*K*_ values for the (111) and (001) orientations are 0.044 erg/cm^2^ and 0.009 erg/cm^2^, respectively. This enhancement of *J*_*K*_ should derive from the more favorable T-domain structure, however, it might also have some contribution due to small structural differences at the (111) interface. For example, it is known that tensile strain on a (001) cubic substrate (as STO) modifies the octahedral rotational pattern in LSMO and, as a consequence, favors 3z^2^-r^2^ orbital occupancy.^52^ The situation could be radically different on (111) where shear strain is not present and bulk a^–^a^–^a^–^ rotation pattern could be preserved and, although a theoretical picture is lacking, the magnetic coupling between LSMO and NiO would be modified. Furthermore, the (111) orientation introduces a more complex stacking sequence, often resulting in a buckled interface with triangular or honeycomb-like symmetry. This altered geometry could enhance spin-Hall effects or enable novel spin textures that may also contribute to the enhanced spin transmission here observed.^53–55^

According to our experimental results in LSMO/NiO/Pt system, the spin current amplification may have different contributions. On the one hand, part of this amplification may be due to greater effective spin transparency at the LSMO/NiO/Pt interface in (111)-oriented heterostructures. The insertion of the NiO spacer layer between LSMO and Pt can improve spin current transmission by mitigating SBF and SML at the LSMO/NiO interface and suppressing magnetic proximity effects in Pt. In spite of the strong similarity, both structurally and chemically, (111)-oriented samples have superior magnetic properties with lower damping and higher Néel temperatures. This suggests a better quality of the “magnetic” interface which may facilitate more efficient spin transport across the interface leading to a larger and $G_{eff}^{↓↑}$ and better spin transparency.

On the other hand, the correlation between the V_ISHE_ signal amplification and the Néel temperature indeed suggests that the enhancement cannot be attributed solely to static improvements in effective spin transparency at the LSMO/NiO/Pt interfaces. Therefore, the amplification must necessarily be related to the dynamics of AF spin correlations in NiO, which are highly temperature-dependent and particularly pronounced near T_N_.^10,11,46,47^ Around this critical point, short-range AF spin fluctuations become more intense and long-lasting, which may facilitate more efficient propagation of spin angular momentum through the NiO layer. This behavior has been reported in several studies, where magnon-mediated spin transport in AF is maximized around T_N_ due to the enhanced magnon population and coherence. In our (111)-oriented samples, which are structurally more favorable for spin-wave propagation due to the more favorable orientation of the Néel vector, this effect is more prominent—leading to a resonant-like enhancement of the spin current transmission and, consequently, the higher V_ISHE_ signal in Pt. Therefore, although the improvement in spin transparency due to the quality and orientation of the interface plays an important role, the observed amplification should critically depend on the dynamic spin transport properties of NiO near T_N_. This reflects a synergistic effect: the (111) orientation provides a structurally favorable path for spin waves due to the more favorable orientation of the Néel vector, and the proximity to T_N_ allows for dynamic amplification through thermally excited spin fluctuations in the AF spacer.

## 3 Conclusions

In this study, we systematically investigate the role of AF materials as insertion barriers and their influence on interfacial properties and spin transport in FM/NM bilayers. To this end, we fabricated epitaxially engineered LSMO/NiO/Pt heterostructures on (001)- and (111)-oriented STO substrates, varying the thickness of the NiO layer. Structural characterization using AFM, XRD and high-resolution STEM confirms that all samples exhibit high crystallinity, atomically smooth surfaces, and sharply defined interfaces. Despite a considerable lattice mismatch (∼7%) between LSMO and NiO, epitaxial growth is achieved through strain relaxation facilitated by interfacial dislocations. Nonetheless, a residual stress remains present, as indicated by an expanded NiO unit cell volume compared to its bulk counterpart, regardless of crystallographic orientation.

Magnetic characterization reveals the presence of EB effects, confirming AF ordering in the NiO layer, with similar blocking temperatures observed for both substrate orientations. Notably, the (111)-oriented heterostructures exhibit significantly improved magnetic and spin transport properties, including higher H_EB_ field, reduced Gilbert damping, longer λ_Sd_, and larger $G_{eff}^{↓↑}$ . To evaluate spin current transmission through the NiO layer, we measured the transverse V_ISHE_ voltage signal induced by ISHE in the Pt layer. At room temperature, the V_ISHE_ signal in (111)-oriented samples is nearly twice as large as that observed in (001)-oriented samples.

Furthermore, the temperature dependence of V_ISHE_ exhibits distinct behavior between the two orientations. In (001)-oriented samples, V_ISHE_ decreases monotonically with decreasing temperature, a trend attributed to enhanced damping in LSMO, stronger AF interactions in NiO, and the reduced resistivity of Pt. In contrast, (111)-oriented samples display a pronounced V_ISHE_ peak that shifts to higher temperatures and decreases in magnitude, from 1.5 μV to 0.29 μV, as NiO thickness increases. A broad temperature range of V_ISHE_ amplification is observed for (111)-oriented samples with NiO thickness up to ∼3 nm. For (001)-oriented samples, however, such amplification is significantly weaker and is only detected near room temperature for the t_NiO_ = 1 nm sample. Below the T_Peak_ ≈ T_N_, V_ISHE_ diminishes progressively due to suppression of incoherent magnons by the onset of long-range AF order, thereby reducing spin transport efficiency. Around T_N_, thermal magnons and spin fluctuations are at their maximum, facilitating enhanced spin conduction. Above T_N_, although short-range spin correlations persist, magnons with wavelengths exceeding the spin correlation length (ξ_AF_ ∼ (|T–T_N_|)^−1/2^) are lost, leading to a gradual decline in the V_ISHE_ signal. The dependence of V_ISHE_ on NiO thickness shows a maximum at approximately 1 nm followed by a quasi-exponential decay, indicating a spin diffusion-limited transport mechanism. However, the short values of λ_Sd_ extracted from our data suggest that spin conduction is primarily governed by thermal magnons and short-range magnetic correlations.^11^ The superior spin conduction efficiency observed in (111)-oriented samples is primarily attributed to a more favorable orientation of the Néel vector and distinct interface symmetry, which together enhance spin-Hall-related effects and support the emergence of novel spin textures. The observed V_ISHE_ signal amplification in the (111)-oriented LSMO/NiO/Pt samples is mainly attributed to a synergistic combination of interfacial and dynamic effects: (i) Enhanced spin transparency due to a reduction of SBF and SML. (ii) Orientation-enhanced spin-wave transport efficiency due to a more favorable orientation of the Néel vector. (iii) Dynamic spin current enhancement mediated by AF fluctuations near T_N_. Collectively, these results underscore the critical role of Néel vector orientation and crystallographic alignment in AF spin transport, offering valuable guidelines for the development of next-generation spintronic devices.

## 4 Experimental Section

A set of LSMO/NiO/Pt trilayers were prepared by using RF sputtering on top of (001) and (111)-oriented STO single-crystal substrates. Fifteen nm thick LSMO films were deposited at 850 °C and 140 mTorr of pressure in an Ar–O_2_ atmosphere. Then, the films were annealed at 850 °C in high-pressure oxygen atmosphere (380 Torr). NiO layers, with t_NiO_ ranging from 0.5 to 10 nm, were deposited by DC sputtering on top of LSMO films at 450 °C and 50 mTorr pressure in an Ar–O_2_ atmosphere from a 0.5 mm thick Ni foil target. Finally, NiO layers were covered with 5 nm thick Pt layers deposited also by DC sputtering at room temperature (RT) and a pressure of 5 mTorr in Ar atmosphere from a Pt foil target.

The microstructural characterization of the samples was performed by using a combination of XRD and STEM techniques. XRD structural characterization was conducted using a Bruker A8 Discover diffractometer and a Bruker D8-Advance diffractometer (equipped with a 2D detector). It reveals that LSMO and NiO grow epitaxially cube-on-cube onto the STO substrate, with epitaxy relations STO(001)//LSMO(001)/NiO(001) and STO(111)//LSMO(111)/NiO(111) (pseudocubic indexation is used throughout for simplicity). The heterostructures demonstrate excellent crystalline quality with no detectable impurity phases. Atomic force microscopy (AFM) topography images reveal that the samples possess exceptionally flat surfaces, with RMS surface roughness values typically around 0.15–0.20 nm. Additionally, the characteristic terraces and step structure of the underlying STO (001) substrates are clearly visible (see Figures S1 and S2).

Local characterization of the heterostructures was conducted using aberration-corrected STEM on a Thermo Fisher Scientific probe-corrected Titan 60–300 microscope. The instrument was operated at 300 kV and featured a high-brightness Schottky field emission gun (X-FEG), a Wien filter monochromator, a CETCOR aberration corrector for the condenser system (by CEOS), and an Ultim Max TLE10 energy-dispersive X-ray spectroscopy (EDS) system from Oxford Instruments. Atomically resolved Z-contrast images were obtained using high-angle annular dark-field (HAADF) imaging. The probe had a convergence semiangle of 24 mrad, resulting in a probe size of 1 Å. STEM spectrum line profiles were acquired by combining HAADF imaging with EDS at a beam current of approximately 200 pA.^56^

The static magnetic properties of the LSMO and LSMO/NiO bilayers were studied using a SQUID magnetometer by Quantum Design. The diamagnetic signal of the substrate and other instrumental contributions for DC magnetization measurement were properly corrected.^57^ The dynamic magnetic properties of the LSMO thin films and LSMO/NiO bilayers were studied by FMR using a commercial broadband coplanar waveguide (CPW) (by NanOsc) inserted into a physical properties measurement system (PPMS by Quantum Design). The transversal voltage signal generated by ISHE in the LSMO/NiO/Pt trilayers was measured by using a 2128 A Keithley nanovoltmeter. This equipment has been programmed to act synchronously with the FMR spectrometer in order to measure both the FMR spectral curves and ISHE voltage signal simultaneously.^58^ A custom-made CPW specifically designed to measure voltage signals in FMR experiments has been employed. The electrical contact with the sample is enabled through two parallel Au ribbons on both sides of the CPW with bumps raised 20 μm above the sample holder surface (see Figure S3).

## Supplementary Material



## Notes

Data corresponding to the atomic force microscopy (AFM) characterization and the description of the experimental setup for the VISHE measurements, high resolution electron microscopy characterization including geometrical phase analysis (GPA) and chemical composition analysis by STEM-EDS, and exhaustive magnetic characterization of the samples (PDF)
