# Supplementary material for: Crystallographic Engineering of Spin Transport in Antiferromagnetic NiO Thin Films
Source: ACS Nano. 2025 Sep 3;19(36):32170–82. doi: 10.1021/acsnano.5c06120 (PMC12445011; doi:10.1021/acsnano.5c06120)
Supplement: Supplementary file 1 [file nn5c06120_si_001.pdf]

Supporting Information

CRYSTALLOGRAPHIC ENGINEERING OF SPIN TRANSPORT IN ANTIFERROMAGNETIC NiO THIN FILMS

Shoulong Chen[1], Alberto Pomar[1], Lluis Balcells[1], Zorica Konstantinovic[2], Bernat Bozzo[1], Carlos Frontera[1], César Magén[3], Narcis Mestres[1] and Benjamin Martinez[1]

[1]Instituto de Ciencia de Materiales de Barcelona. ICMAB-CSIC. Campus Universitario UAB, Bellaterra 08193, Spain.
[2]Center for Solid State Physics and New Materials, Institute of Physics Belgrade, University of Belgrade, Belgrade 11000, Serbia.
[3]Instituto de Nanociencia y Materiales de Aragón (INMA), CSIC-Universidad de Zaragoza, 50009 Zaragoza, Spain.

Corresponding author:
Benjamin Martinez. E-mail: ben.martinez@icmab.es

Microstructural characterization:

Atomic force microscopy (AFM) topography images reveal that the samples possess exceptionally flat surfaces, with RMS surface roughness values typically around 0.15-0.20 nm indicating that the heterostructures grown on both types of substrates exhibit atomic-level flatness.

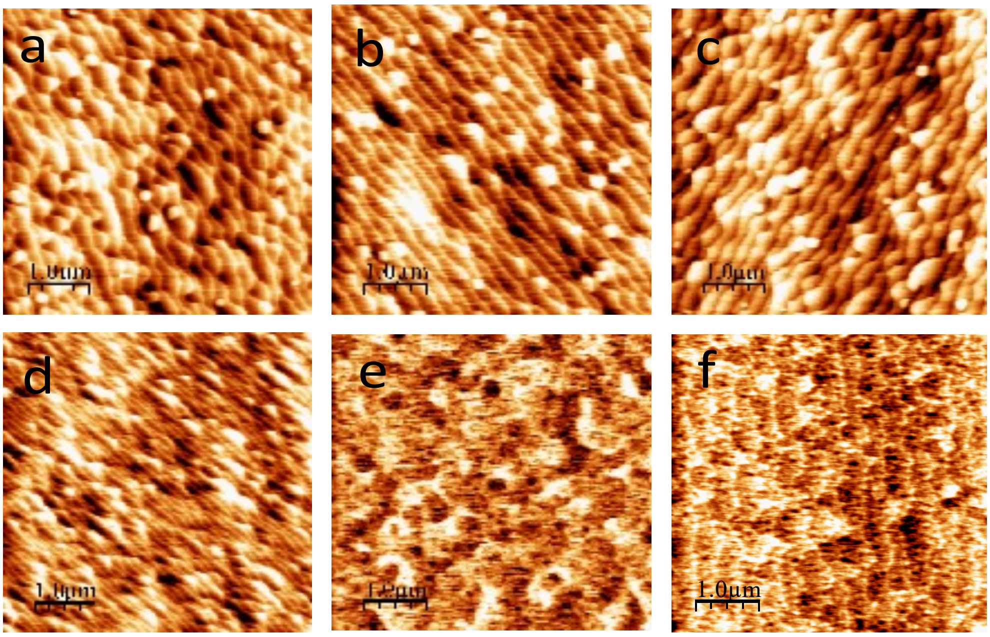

Figure S1: AFM images of the STO(001)//LSMO/NiO(t)/Pt heterostructures corresponding to NiO thicknesses t= 1 nm, 1.5 nm, 2 nm, 3 nm, 5 nm, and 10 nm (a to f). Their RMS surface roughness values are 0.15 nm, 0.13 nm, 0.15 nm, 0.14 nm, 0.20 nm, and 0.14nm, respectively.

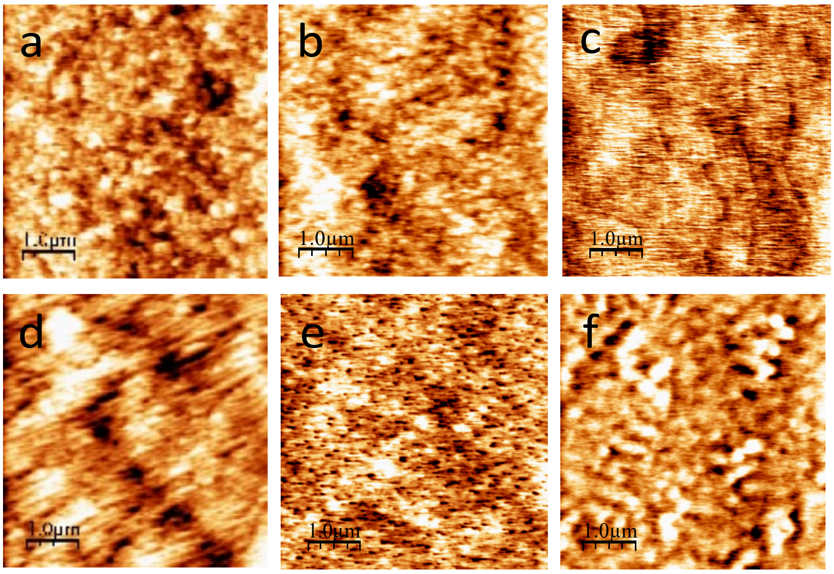

Figure S2: AFM images of the STO(111)//LSMO/NiO(t)/Pt heterostructures corresponding to NiO thicknesses t= 1 nm, 1.5 nm, 2 nm, 3 nm, 5 nm, and 10 nm (a to f). Their RMS surface roughness values are 0.14 nm, 0.14 nm, 0.13 nm, 0.09 nm, 0.15 nm, and 0.17 nm, respectively.

ISHE measurements and expected sign for the transverse voltage signal V_{ISHE}:

The experimental setup used for ISHE measurements is shown in **Fig. S3b**. As described in our previous work in this topic,[1,2] two 30-nm thick stripes of gold were deposited on both sides of each sample in order to make a proper electrical contact with the FMR sample holder. At the same time, a Keithley 2182A nanovoltmeter was synchronized with the broadband FMR spectrometer to acquire the transverse voltage signal.

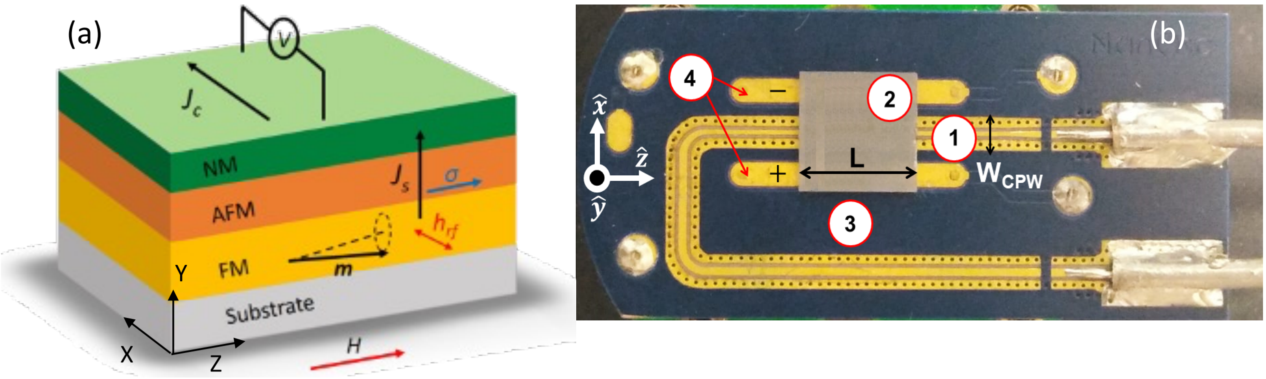

Figure S3: (a) Schematic picture of the geometry of the samples used for spin pumping and ISHE transverse voltage signal measurements. (b) Top view picture of the actual ISHE measurement system. The numbers correspond to the following parts: (1) CPW with a total width W_{CPW} = 1.4 mm (including the RF signal active line in the middle and the two ground lines on both sides). (2) The sample placed upside down on top of the CPW with a total length L = 5 mm. (3) Insulating layer. (4) Contact pads with bumps raised 20 µm above the insulating layer for making electrical contact with the sample by means of Au contacts. The HI and LO ends, represented by + and − signs, respectively, define the polarity of the nanovoltmeter. Finally, Kapton tape is used to fix and tighten the sample onto the CPW (not shown). This sample holder is the "CPW PPMS IP ISHE" model produced by NanOsc Instruments.[3]

To understand why the expected ISHE voltage sign should be positive in our experimental setup, we refer to the coordinate system in Fig. S3. The applied magnetic field aligns with the z-axis, meaning the spin polarization vector $\hat{\boldsymbol{\sigma}}$, parallel to the quantization axis, also points in the z-direction. The spin current $\boldsymbol{j}_s$ flows from LSMO into Pt, directed along ***-y*** direction. Since the ISHE-induced transverse charge current follows $\boldsymbol{j}_c = \theta_{\mathrm{SH}} \boldsymbol{j}_s \times \hat{\boldsymbol{\sigma}}$, it flows in the ***-x***-direction, leading to charge accumulation on both sides of the sample. This accumulation induces an opposing electric field along **+*x*,** resulting, finally, in an ISHE-induced transverse voltage drop. By definition, the measured voltage is $V \equiv V^{+} - V^{-}$, where the + and – symbols (shown in Fig. S3) indicate contact polarity. The voltage drop due to ISHE is defined by the line integral:

$$V^{\mathrm{ISHE}} = -\int_a^b \langle \boldsymbol{E}^{\mathrm{ISHE}} \rangle d\boldsymbol{x}$$

Recalling that $V \equiv V^{+} - V^{-}$, then the integration limits are set to be $a = +W_{\mathrm{CPW}}/2$ and $b = -W_{\mathrm{CPW}}/2$. As a consequence, the result of the integral will be negative. However, the negative sign in front ensures that the overall ISHE-induced voltage remains positive.

LSMO/NiO interfacial features:

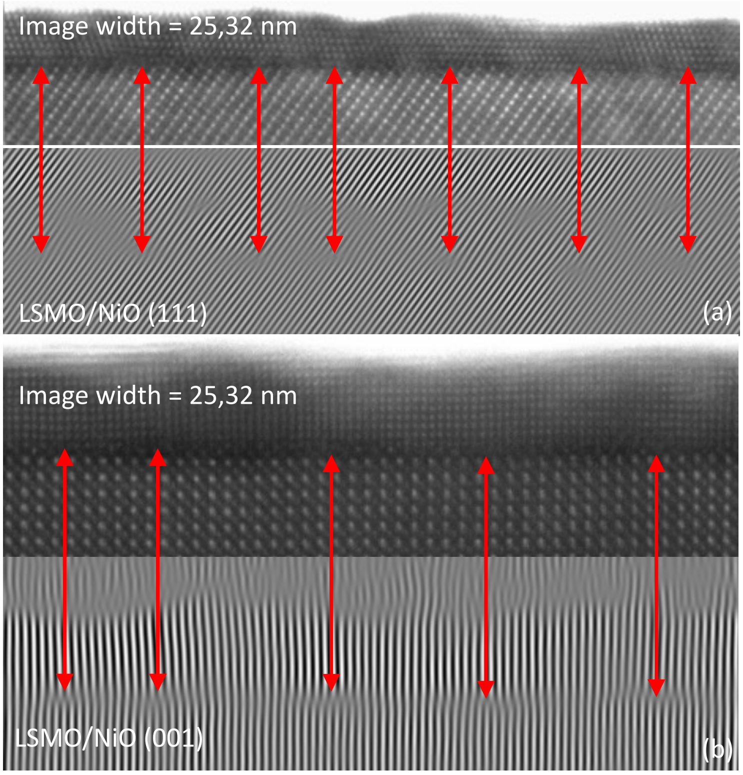

Figure S4: (a) Top: atomic-resolution STEMHAADF image of the interface LSMO(15 nm)/NiO (2 nm) region, films grown on (111)-STO. Bottom: Fourier filtered image, obtained by [100] diffraction reflection from the STO substrate and NiO epilayer, outlining the common (020) planes between the film and the substrate. (b) Top: atomic-resolution STEM-HAADF image of the interface LSMO(15nm)/NiO (2 nm) region, films grown on (111)-STO. Bottom: Fourier filtered image, obtained by [001] diffraction reflection from the STO substrate and NiO epilayer, outlining the common (002) planes between the film and the substrate.

At the LSMO/NiO interface, the typical effect of a film growing largely epitaxially, albeit with some mosaicity, but without significant strain, is observed. As a result, misfit dislocations appear directly at the interface (see **Fig. S4**). The images have been filtered by selecting the spots corresponding to the most suitable planes for observing this mismatch. According to the nominal lattice parameters of both materials, if LSMO was to exactly adopt the substrate lattice (3.905 Å), an additional LSMO plane would appear approximately every 14 NiO planes (4.177 Å), corresponding to a spacing of about 5,8 nm. However, a slightly larger number of dislocations is observed, with an average dislocation spacing of 3.8 ± 0.8 Å for the (111)-oriented films and 5.1 ± 1.2 Å for the (001)-oriented ones.
Geometric Phase Analysis (GPA) was employed to evaluate strain and deformation in High-Angle Annular Dark Field (HAADF) Scanning Transmission Electron Microscopy (STEM) images of STO//LSMO/NiO/Pt heterostructures grown along the (001) and (111) crystallographic directions. GPA offers both visual and quantitative insights into the strain state of the NiO layer on LSMO. The analysis indicates tensile strain in both the x and y directions, consistent with the unit cell expansion observed in XRD measurements for a thicker NiO layer.

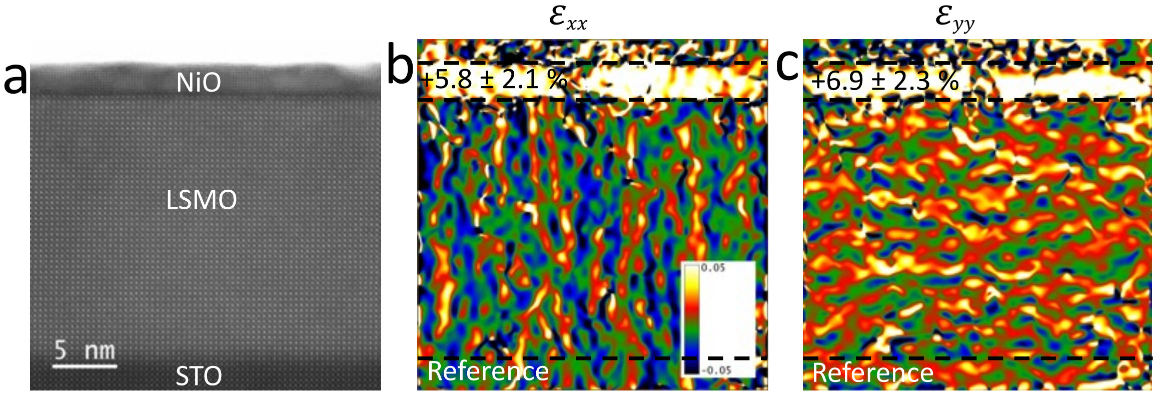

Figure S5: (a) Atomic-resolution cross-sectional HAADF-STEM images of the STO(001)//LSMO/NiO(2nm)/Pt sample. GPA study: (b) In-plane and (c) out-of-plane lattice strain maps are shown. The corresponding deformation parameters: ε_{xx}~ +5.8% ± 2.1% and ε_{yy}~6.9% ± 2.3% respect to the STO were determined.

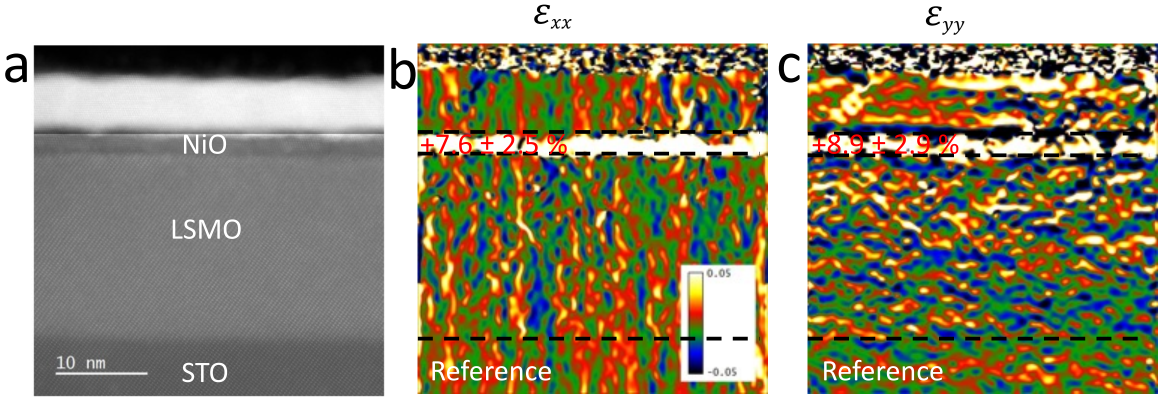

Figure S6: (a) Atomic-resolution cross-sectional HAADF-STEM images of the STO(111)//LSMO/NiO(2nm)/Pt sample. GPA study: (b) In-plane and (c) out-of-plane lattice strain maps are shown. The corresponding deformation parameters: ε_{xx}~ +7.6% ± 2.5% and ε_{yy}~8.9% ± 2.9% respect to the STO were determined.

A more in-depth characterization of the LSMO/NiO interface can be obtained through chemical composition analysis by STEM-EDS. **Fig. S7** shows the compositional profiles corresponding to two STO//LSMO/NiO/Pt samples grown in the crystallographic directions (001) and (111) with a nominal NiO layer thickness of 2 nm. The figure shows that the heterostructures are of high quality and have abrupt interfaces with a relatively low interdiffusion of atomic species.

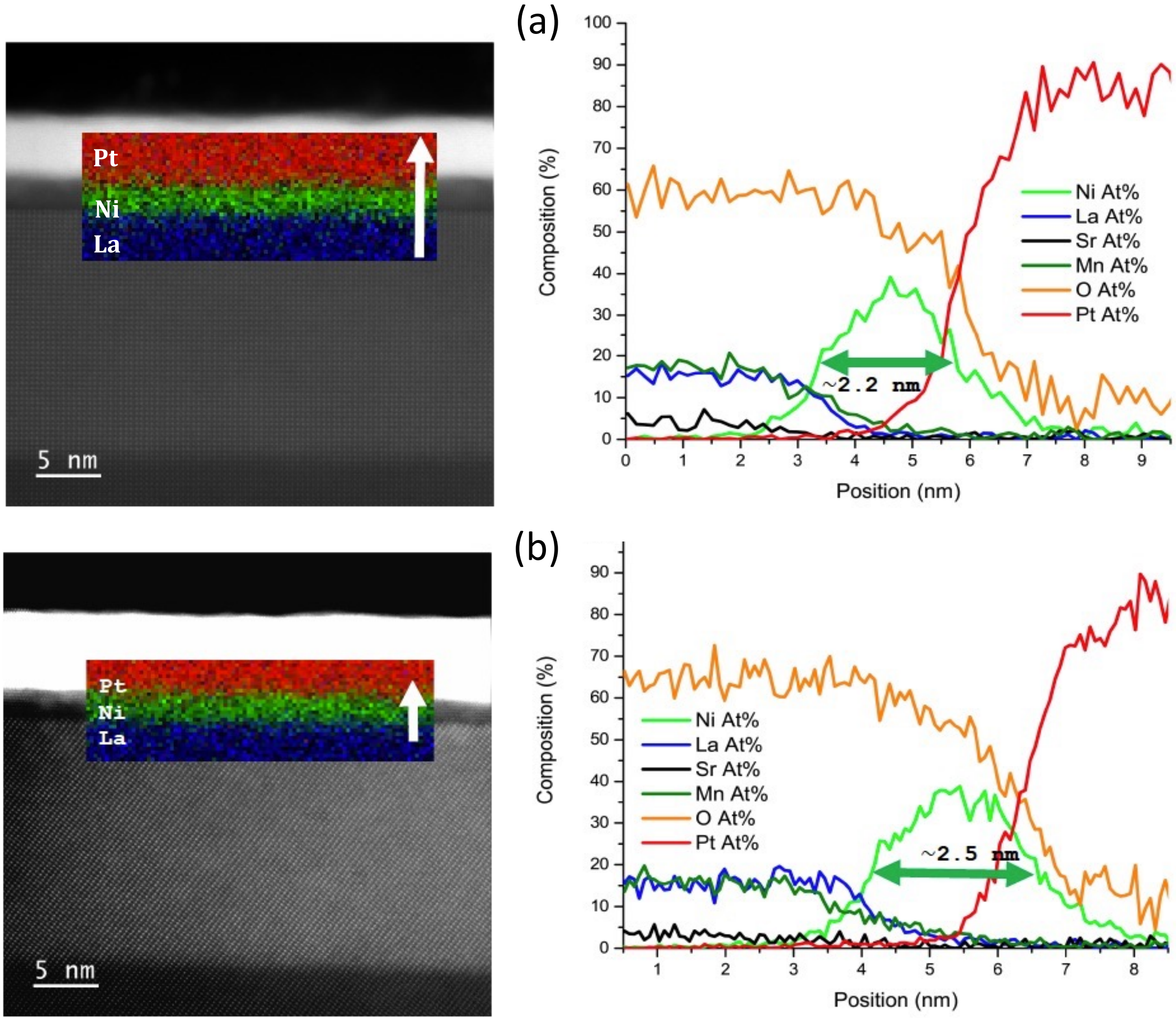

Figure S7: STEM-EDS chemical analysis of the LSMO/NiO/Pt trilayer for two samples with different crystallographic orientation: (001)-oriented (a) and (111)-oriented (b) with the same nominal NiO layer thickness (2 nm). HAADF reference images are shown on the left-hand side and the corresponding chemical line profiles, obtained by horizontal integration of ~10 nm of the EDS maps across the interface, are shown in the right-hand side.

DC magnetic properties:

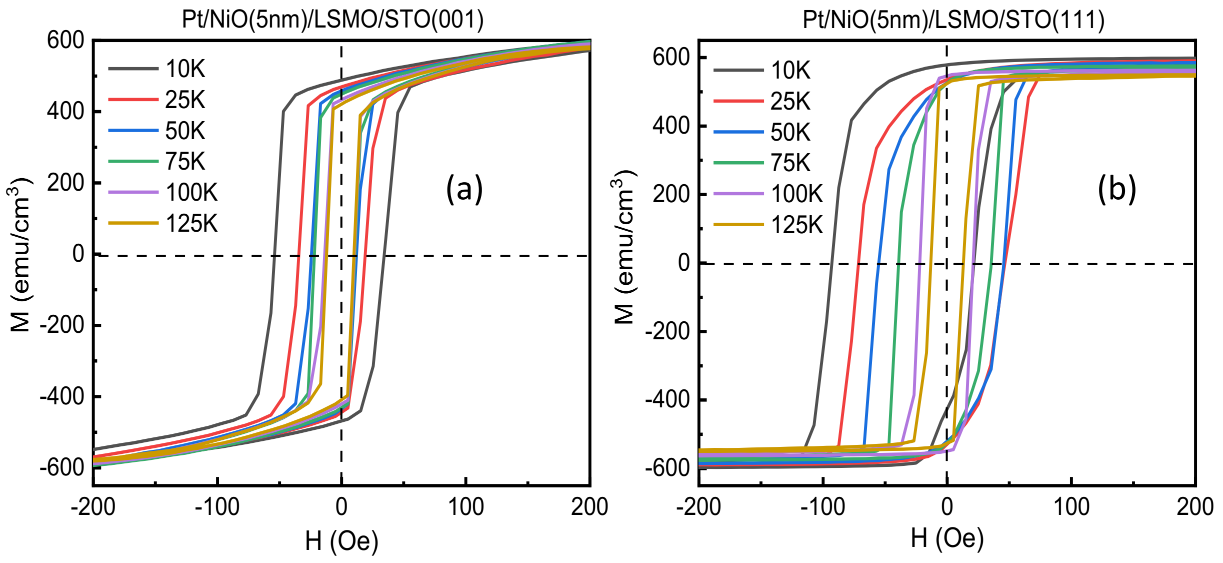

Figure S8: M(H) loops showing the temperature dependence of the exchange bias field in STO//LSMO/NiO(5 nm)/Pt heterostructures for different crystallographic growth directions: (a) (001) and (b) (111).

Dynamic magnetic properties of LSMO thin films and LSMO/NiO/Pt trilayers

The quality of the LSMO layers was evaluated by measuring FMR linewidths (ΔH) before the deposition of the NiO and Pt layers. Ensuring that the LSMO layers exhibit uniform ΔH is crucial for obtaining reliable comparisons of V_{ISHE} signals across samples with different NiO layer thicknesses.

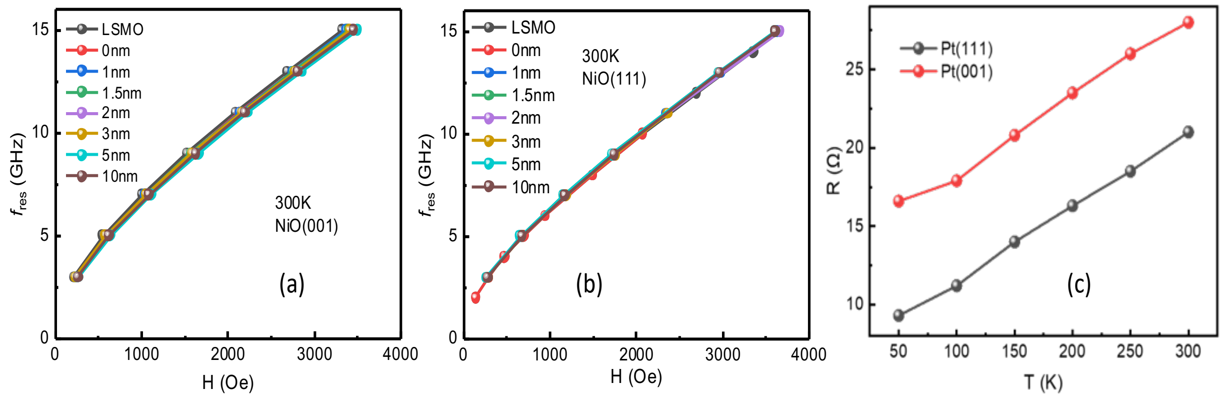

Figure S9: Dependence of the resonance frequency on the resonance field for STO//LSMO/NiO/Pt heterostructures grown along the (001) (a) and (111) (b) crystallographic directions as a function of the NiO layer thickness. The fits are made according to Eq. 1 in the main text. (c) Transverse resistance of Pt thin films grown along (111) and (001) crystallographic orientations as a function of temperature.

Spin conduction process:

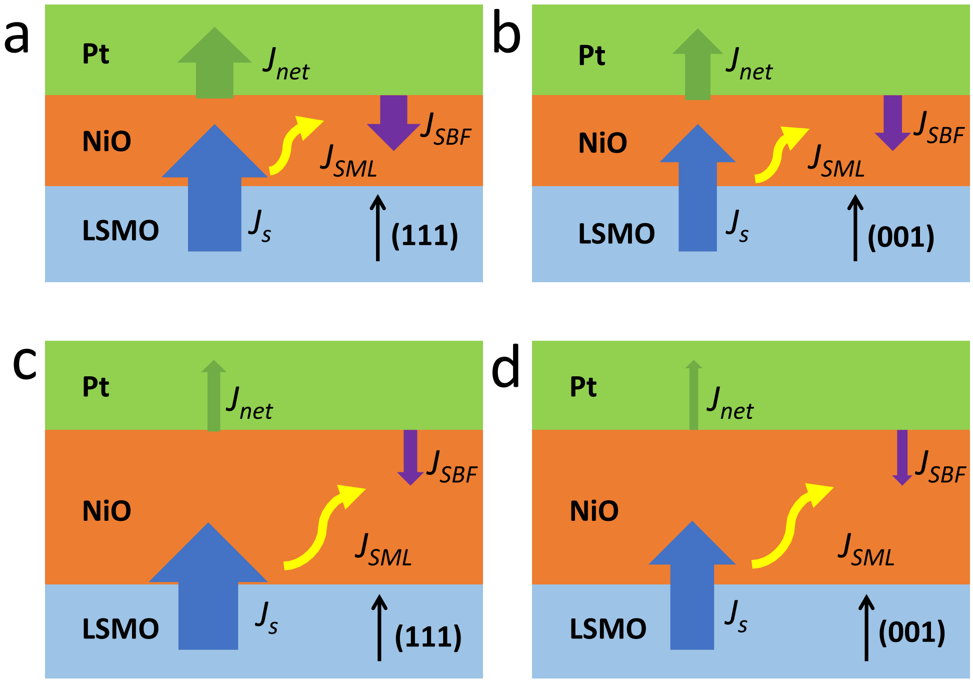

Figure S10: In agreement with the data shown in **Fig. 6** of main text, a transport balance emerges at a specific NiO thickness, where the V_{ISHE} is maximized due to the interplay of spin current transmission, spin memory loss (SML), and spin backflow (SBF). Around 1–2 nm of NiO, (**a**) and (**b**) both the transmitted spin current, J_{net}, (reaching the Pt layer) and the SBF component are at their peak. As the NiO layer thickens, spin current transmission through the AFM layer gradually diminishes—due to enhanced absorption and scattering (SML) —thus reducing the spin current that reaches the Pt layer. For instance, at an NiO thickness of around 10 nm (**c** and **d**), the total spin current is largely governed by SML, with minimal contribution of SBF. The size of the arrows is intended to reflect the relative intensity of each term.

Figure S10 shows a schematic illustration of spin current conduction across the NiO layer. The pure spin current, J_S, is generated via spin pumping in the LSMO layer and injected into the adjacent NiO layer. As this spin current propagates through the NiO, part of it is dissipated due to spin scattering processes—this loss is identified as spin memory loss (J_{SML}). Additionally, a portion of the spin current (J_{SBF}) can be reflected at the NiO/Pt interface, resulting in spin backflow. Consequently, the net spin current that reaches the Pt layer is given by: $J_{net} = J_S - J_{SML} - J_{SBF}$. The relative impact of spin memory loss and spin backflow depends on the NiO layer thickness as well as the interfacial properties at both the LSMO/NiO and NiO/Pt interfaces. For thin NiO layers, J_{SBF} tends to dominate over J_{MSL}. However, as the NiO thickness increases—especially beyond 6–8 nm— J_{SBF} becomes negligible, and the primary cause of spin current reduction is spin memory loss. This balance is influenced not only by t_{NiO} but also by the crystallographic orientation and interfacial quality. In (111)-oriented NiO, the Néel vector is better aligned with the injected spin current, leading to enhanced interfacial spin mixing conductance and more efficient spin current injection into the NiO layer. Moreover, the (111) orientation supports a longer spin diffusion length, enabling spin currents to propagate larger distances into the NiO. In contrast, the (001) orientation features a less favorable alignment of the Néel vector with the spin current, resulting in reduced spin

transmissivity and greater backflow into LSMO. In addition, spin currents suffer from increased attenuation due to a shorter spin diffusion length. As a result, the net spin angular momentum reaching the Pt layer is significantly greater in the (111)-oriented samples, reflected in stronger ISHE signals. Although spin mixing conductance tends to increase with t_{NiO} in both orientations, spin memory loss leads to a diminishing J_{net} at larger thicknesses. Our experimental data align with this interpretation, consistently showing that (111)-oriented samples exhibit higher spin mixing conductance, extended spin diffusion lengths, and stronger ISHE responses compared to the (001)-oriented samples, which exhibit weaker and more rapidly decaying spin signals.

NiO AF domain structure:

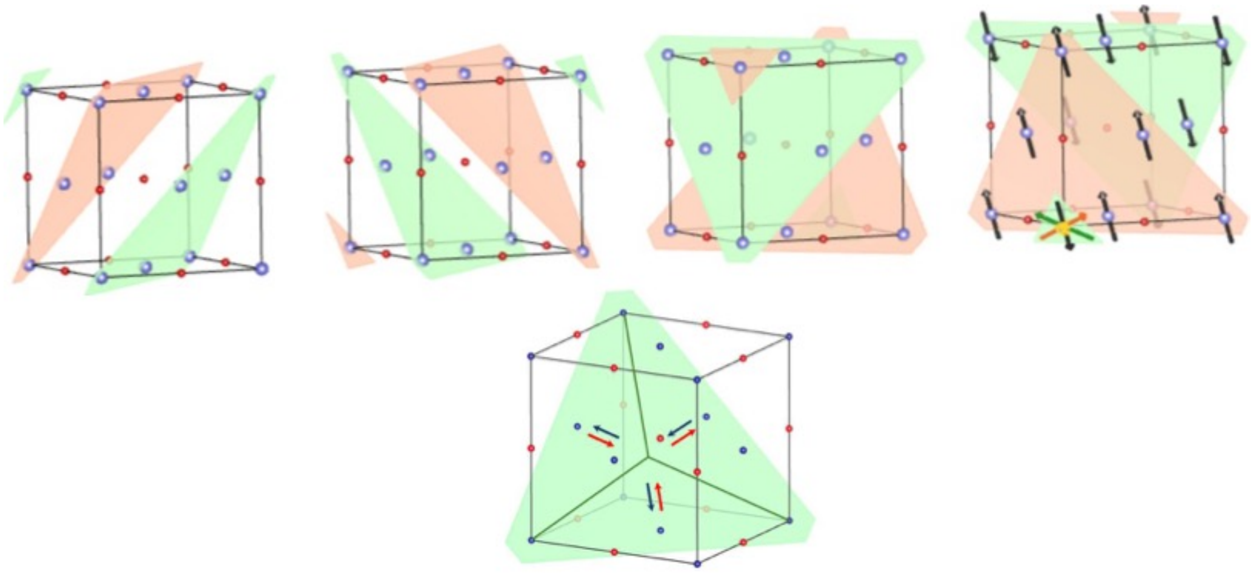

Figure S11: Schematic illustration of the four possible orientations of the {111} planes in NiO, where spins couple ferromagnetically, leading to the formation of four distinct T-domains. Additionally, the diagram indicates the three possible moment orientations within each T-domain, giving rise to three corresponding S-domains is shown (bottom cube).

References:

[1] S. Martín-Rio, C. Frontera, A. Pomar, Ll. Balcells & B. Martinez. Scientific reports, 12, 1 (2022).

[2] S. Martin-Rio, A. Pomar, C. Frontera, H. Wang, R. Manzorro, C. Magén, Ll. Balcells, N. Mestres and B. Martinez. J. Mater. Chem. C. 10, 5914–5921 (2022)

[3] NanOsc Instruments. Wave Guides. 2021. url: http://www.nanosc.se/wave- guides-cpws.html.
